# Ruthenium(III) Chloride Catalyzed Acylation of Alcohols, Phenols, and Thiols in Room Temperature Ionic Liquids

**DOI:** 10.3390/molecules14093528

**Published:** 2009-09-10

**Authors:** Zhiwen Xi, Wenyan Hao, Pingping Wang, Mingzhong Cai

**Affiliations:** 1 Department of Chemistry, Jiangxi Normal University, Nanchang 330022, China; E-mails: xizhiwen1983@163.com (Z.X.); wenyanhao@tom.com (W.H.); 2 Department of Chemistry, Jiujiang University, Jiujiang 332000, China; E-mail: wpp9090@yahoo.com.cn (P.W.)

**Keywords:** acylation, ruthenium, ionic liquid, green chemistry

## Abstract

Ruthenium(III) chloride-catalyzed acylation of a variety of alcohols, phenols, and thiols was achieved in high yields under mild conditions (room temperature) in the ionic liquid 1-butyl-3-methylimidazolium hexafluorophosphate ([bmim][PF_6_]). The ionic liquid and ruthenium catalyst can be recycled at least 10 times. Our system not only solves the basic problem of ruthenium catalyst reuse, but also avoids the use of volatile acetonitrile as solvent.

## Introduction

The development of environmentally friendly synthetic procedures has become a major concern throughout the chemical industry due to continuing depletion of natural resources and growing environmental awareness [1,2,3,4]. One of the prime concerns of industry and academia is the search for replacements for environmentally damaging organic solvents used on a large scale, especially those which are volatile and difficult to contain. Room temperature ionic liquids, especially those based upon the 1,3-dialkylimidazolium cation, have attracted growing interest in the last few years [5,6,7,8,9]. They offer a possible alternative and ecologically sound medium compared to conventional organic solvents, as they are non-volatile, recyclable, thermally robust and excellent solvents for a wide range of organic and inorganic materials. Furthermore, their high compatibility with transition metal catalysts and limited miscibility with common solvents, enables easy product and catalyst separation with the retention of the stabilized catalyst in the ionic phase [10]. These and related ionic liquids have been successfully applied to hydrogenations [11], alkene dimerizations [12], Friedel-Crafts reactions [13,14], Diels-Alder reactions [15,16,17], Heck reactions [18,19,20], Bechmann condensations [21,22], Suzuki reactions [23,24,25,26], Baylis-Hillman reactions [27,28], Stille reactions [29], and Sonogashira reactions [30].

The acylation of alcohols, phenols, and thiols is a common practice in organic synthesis [31]. In these reactions, acid chlorides or anhydrides are often used as the acyl source in the presence of amine bases such as triethylamine, pyridine, or DMAP [31]. Some methods employing Bu_3_P [32], Sc(OTf)_3_ [33,34], Sc(NTf_2_)_3_ [35], TMSOTf [36,37], Cu(OTf)_2_ [38], InCl_3_ [39], In(OTf)_3_ [40], Bi(OTf)_3_ [41,42], and clays [43] have also been reported. However, many of these methods have some drawbacks such as low yields, long reaction times, harsh reaction conditions, use of hazardous materials (e.g., DMAP is highly toxic, Bu_3_P is flammable and air sensitive), and the use of expensive [33,34,35,36,37,40,41,42] and/or not readily available reagents [33,34,41,42]. Some of the reported methods work well on primary or secondary alcohols only and fail to protect tertiary alcohols or less reactive phenols. Recently, Kanta De described ruthenium chloride catalyzed acylation of alcohols, phenols, thiols, and amines [44]. The reaction proceeds in the presence of 5 mol% RuCl_3_ in acetonitrile at room temperature. Although this method has the major advantages of high yields, short reaction times, and compatibility with other protecting groups, the expensive homogeneous ruthenium catalyst can’t be recovered and reused and acetonitrile is volatile and toxic. These disadvantages have so far precluded its practical applications. In this paper, we describe the ruthenium-catalyzed acylation of alcohols, phenols, and thiols in room temperature ionic liquids.

## Results and Discussion

**Scheme 1:**
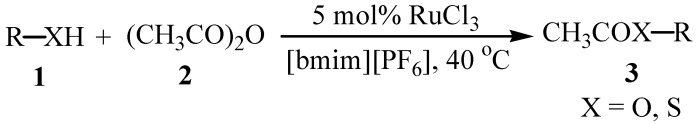
RuCl_3_-catalyzed acylation reaction in [bmin][PF_6_].

As a suitable solvent for the acylation of alcohols, phenols, and thiols, the moisture stable and readily available 1-butyl-3-methylimidiazolium hexafluorophosphate ([bmim][PF_6_]) was chosen. The reaction of benzyl alcohol with acetic anhydride in the presence of 5 mol% RuCl_3_ in [bmim][PF_6_] at 40°C afforded the desired benzyl acetate in 92% yield in 10 min. The acylation of a variety of alcohols, phenols, and thiols with acetic anhydride in the presence of 5 mol% RuCl_3_ was investigated (Scheme 1), the experimental results are showed in Table 1. As shown in the Table, primary and secondary alcohols underwent the acetylation reactions in excellent yields (entries 1-4). Interestingly, tertiary alcohols can also be acetylated smoothly in high yields without any side products being observed (entry 5). Phenols are less reactive than alcohols toward acylation reactions but nevertheless they underwent acylation smoothly. Compared with the acylation of primary alcohols, the acylation of secondary or tertiary alcohols and phenols needed longer reaction times. The ionic liquid, [bmim][PF_6_], has the advantage of increase in reaction rate over acetonitrile as the solvent. For example, the acetylation reaction of 2-naphthol in [bmim][PF_6_] within 2.5 h gave the corresponding acetylated product in 93% yield (entry 14), the same reaction in acetonitrile required 10 h to go to completion, affording the acetylated product in 91% yield [44].

**Table 1:** RuCl_3_-catalyzed acylation of alcohols, phenols, and thiols in [bmim][PF_6_].^ a^

Entry	Substrate	Time	Product	Yield (%)^b^
1	PhCH_2_OH	10 min	**3a**	92
2	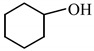	1.5 h	**3b**	91
3	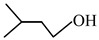	10 min	**3c**	90
4	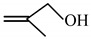	30 min	**3d**	93
5	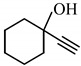	3 h	**3e**	87
6	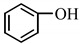	2 h	**3f**	89
7	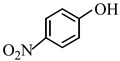	2 h	**3g**	98
8	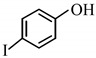	1.8 h	**3h**	92
9	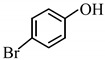	2 h	**3i**	90
10	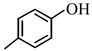	2 h	**3j**	95
11	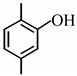	2.5 h	**3k**	88
12	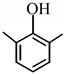	3 h	**3l**	90
13	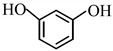	3 h	**3m**	90
14	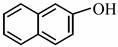	2.5 h	**3n**	93
15	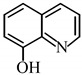	2 h	**3o**	98
16	PhSH	1 h	**3p**	95
17	*n*-BuSH	35 min	**3q**	91
18	4-ClC_6_H_4_SH	1.5 h	**3r**	90
19	4-CH_3_C_6_H_4_SH	2 h	**3s**	92

^a ^Reaction was carried out with 1 mmol of substrate, 1.2 equiv of Ac_2_O, and 5 mol% RuCl_3 _in 1.5 mL of [bmim][PF_6_] at 40 °C under Ar. ^b ^Yields refer to pure isolated products.

To explore generality and scope further, the RuCl_3_-catalyzed acylation in [bmim][PF_6_] was examined using other functionally and sterically diverse alcohols or phenols, as listed in Table 1. When 2,6-dimethylphenol was subjected to the present method, acylation took place smoothly in excellent yield (Table 1, entry 12). Similarly, sterically hindered substrates, (entries 5 and 11) were acetylated to give a high yield of product. Resorcinol underwent acetylation smoothly to give benzene-1,3-diyl diacetate in 90% yield (entry 13, Table 1). This method can be extended to acetylation reactions of thiols, and a variety of thiols underwent acylation smoothly in excellent yields (entries 16-19). Although metal triflates (Otera [41,42] type and others [33,34,36,37,38]) have recently been reported as Lewis acid catalysts for acetylation reactions, these catalysts are expensive and commercially unavailable (in some cases). The strong Lewis acid character of metal triflates also makes them unsuitable in use with acid-sensitive substrates such as 1-ethynyl-1-cyclohexanol, necessitating the use of excess acetic anhydride and/or low temperatures (0 to –20 °C) to suppress competitive side reactions. Thus, 1-ethynyl-1-cyclohexanol was acetylated by Sc(OTf)_3_ at –20 °C using three equivalents of acetic anhydride, while Bi(OTf)_3_-catalyzed acetylation of the same compound required 10 equiv of acetic anhydride. The RuCl_3_-catalyzed acetylation of 1-ethynyl-1-cyclohexanol in [bmim][PF_6_] at 40 °C requires only 1.2 equiv of acetic anhydride. All the acetylated products were characterized by IR, ^1^H-NMR, ^13^C-NMR.

Isolation of the acetylated products from the [bmim][PF_6_] reaction mixtures can be conveniently achieved by extraction with diethyl ether three times. To evaluate the possibility of recycling the ionic liquid and ruthenium catalyst used in the reaction, benzyl alcohol and acetic anhydride were allowed to react in [bmim][PF_6_] at 40 °C for 10 min and then the product was extracted with diethyl ether for three times affording a cleaned, ionic liquid catalytic solution. After the recovered ionic liquid containing ruthenium catalyst was concentrated *in vacuo* (5.0 torr/r.t. for 1 hour), a second amount of reactants were added and the process was repeated up to 10 times. It seems that there was no effect on the rate and yield of the reaction during 1-10 cycles (Table 2), a result that is important from a practical point of view.

**Table 2:** Ionic liquid and catalyst recycling in the acetylation of benzyl alcohol.^ a^

Cycle	Yield (%)^b^	Cycle	Yield (%)^b^
1	92	6	90
2	91	7	91
3	92	8	90
4	90	9	89
5	91	10	89

^a ^Reaction was conducted under the conditions of 1.0 mmol of benzyl alcohol and 1.2 mmol of acetic anhydride in the presence of RuCl_3 _(5 mol%) in 1.5 mL of [bmim][PF_6_] at 40 °C for 10 min under Ar. ^b^ Isolated yield based on the benzyl alcohol used.

## Experimental

### General

All chemicals were of reagent grade and used as purchased. ^1^H-NMR (400 MHz) spectra and ^13^C-NMR (100 MHz) spectra were recorded on a Bruker AC-P400 spectrometer using CDCl_3_ as the solvent; TMS was the internal standard. IR spectra were determined on an FTS-185 instrument as neat films. All reactions were carried out in pre-dried glassware (150 °C, 4 h) cooled under a stream of dry Ar.

### Typical procedure for the acetylation of benzyl alcohol in ionic liquids

Into a two-necked flask equipped with a magnetic stirring bar were placed ruthenium chloride (0.05 mmol), benzyl alcohol (1.0 mmol), acetic anhydride (1.2 mmol), and [bmim][PF_6_] (1.5 mL) under an Ar atmosphere. The mixture was stirred at 40 °C for 10 min, then cooled to 25 °C and extracted with diethyl ether (3 × 10 mL). The recovered ionic liquid containing ruthenium catalyst was concentrated *in vacuo* (5.0 torr/r.t. for 1 h) and reused in the next run. The combined ether extracts were washed with saturated NH_4_Cl solution (10 mL), 1 N NaHCO_3_ solution (10 mL), and brine (10 mL), dried over MgSO_4_, and concentrated under reduced pressure to give an oil, which was purified by SiO_2_ column chromatography (eluent(s): hexane/EtOAc, 19:1).

*Benzyl acetate* (**3a**) [44]: Oil. ^1^H-NMR: δ 7.43-7.34 (m, 5H), 5.14 (s, 2H), 2.13 (s, 3H); ^13^C-NMR: δ 170.94, 135.94, 128.62, 128.33, 128.31, 66.35, 21.07; IR: ν (cm^-1^) 3035, 2956, 1742, 1498, 1455, 1228, 1027, 736, 698.

*Cyclohexyl acetate* (**3b**) [44]: Oil. ^1^H-NMR: δ 4.76-4.71 (m, 1H), 2.04 (s, 3H), 1.87-1.34 (m, 10H); ^13^C-NMR: δ 170.64, 72.69, 31.63, 25.35, 23.80, 21.44; IR: ν (cm^-1^) 2937, 1737, 1486, 1452, 1240, 1045, 908, 734.

*i-Pentyl acetate* (**3c**) [44]: Oil. ^1^H-NMR: δ 4.09 (t, *J* = 6.8 Hz, 2H), 2.05 (s, 3H), 1.72-1.64 (m, 3H), 0.94 (d, *J* = 7.2 Hz, 6H); ^13^C-NMR: δ 171.25, 63.15, 37.30, 25.05, 22.44, 21.02; IR: ν (cm^-1^) 2961, 1743, 1466, 1367, 1234, 1123.

*Methallyl acetate* (**3d**): Oil. ^1^H-NMR: δ 4.98 (s, 1H), 4.93 (s, 1H), 4.50 (s, 2H), 2.10 (s, 3H), 1.76 (s, 3H); ^13^C-NMR: δ 170.74, 139.90, 112.88, 67.74, 20.32, 14.15; IR: ν (cm^-1^) 2959, 1741, 1454, 1375, 1232, 1027, 912, 734; Anal. Calc. for C_6_H_10_O_2_: C, 63.13; H, 8.83. Found: C, 62.89; H, 8.62%.

*1-Ethynylcyclohexyl acetate* (**3e**) [44]: Oil. ^1^H-NMR: δ 2.61 (s, 1H), 2.15-2.11 (m, 2H), 2.05 (s, 3H), 1.88-1.80 (m, 2H), 1.66-1.59 (m, 4H), 1.56-1.49 (m, 1H), 1.39-1.30(m, 1H); ^13^C-NMR: δ 169.30, 83.65, 75.10, 74.22, 36.90, 25.08, 22.44, 21.94; IR: ν (cm^-1^) 3287, 2938, 2862, 2112, 1745, 1447, 1367, 1229, 1025.

*Phenyl acetate* (**3f**) [44]: Oil. ^1^H-NMR: δ 7.40 (t, *J* = 8.0 Hz, 2H), 7.25 (t, *J* = 7.6 Hz, 1H), 7.11 (d, *J* = 8.0 Hz, 2H), 2.31 (s, 3H); ^13^C-NMR: δ 169.48, 150.73, 129.44, 125.83, 121.59, 21.13; IR: ν (cm^-1^) 3064, 2927, 1765, 1594, 1493, 1370, 1193, 748, 691.

*4-Nitrophenyl acetate* (**3g**) [44]: Oil. ^1^H-NMR: δ 8.28 (d, *J* = 8.8 Hz, 2H), 7.29 (d, *J* = 8.8 Hz, 2H), 2.36 (s, 3H); ^13^C-NMR: δ 168.46, 155.36, 146.15, 125.24, 122.48, 21.16; IR: ν (cm^-1^) 3113, 1762, 1591, 1521, 1487, 1348, 1193, 917, 868.

*4-Iodophenyl acetate* (**3h**) [45]: Oil. ^1^H-NMR: δ 7.67 (d, *J* = 8.8 Hz, 2H), 6.87-6.83 (m, 2H), 2.27 (s, 3H); ^13^C-NMR: δ 169.02, 150.53, 138.48, 123.81, 89.88, 21.11; IR: ν (cm^-1^) 3066, 2925, 1761, 1579, 1481, 1368, 1195, 1009, 908, 840.

*4-Bromophenyl acetate* (**3i**) [44]: Oil. ^1^H-NMR: δ 7.50 (d, *J* = 8.4 Hz, 2H), 7.00 (d, *J* = 8.4 Hz, 2H), 2.31 (s, 3H); ^13^C-NMR: δ 169.19, 149.67, 132.49, 123.45, 118.93, 21.12; IR: ν (cm^-1^) 3070, 2936, 1762, 1585, 1485, 1369, 1197, 1012, 907, 842.

*4-Methylphenyl acetate* (**3j**) [46]: Oil. ^1^H-NMR: δ 7.09 (d, *J* = 8.4 Hz, 2H), 6.89 (d, *J* = 8.4 Hz, 2H), 2.26 (s, 3H), 2.21 (s, 3H); ^13^C-NMR: δ 169.85, 148.42, 135.53, 129.99, 121.28, 21.16, 20.91; IR: ν (cm^-1^) 3035, 2926, 1763, 1508, 1369, 1195, 1018, 909, 845.

*2,5-Dimethylphenyl acetate* (**3k**) [47]: Oil. ^1^H-NMR: δ 7.13 (d, *J* = 7.6 Hz, 1H), 6.97 (d, *J* = 8.0 Hz, 1H), 6.84 (s, 1H), 2.33 (s, 3H), 2.32 (s, 3H), 2.16 (s, 3H); ^13^C-NMR: δ 169.44, 149.17, 136.93, 130.87, 126.90, 126.81, 122.40, 20.92, 20.85, 15.76; IR: ν (cm^-1^) 2926, 1768, 1578, 1510, 1369, 1214, 1110, 893, 810.

*2,6-Dimethylphenyl acetate* (**3l**) [44]: Oil. ^1^H-NMR: δ 7.09-7.04 (m, 3H), 2.32 (s, 3H), 2.15 (s, 6H); ^13^C-NMR: δ 168.85, 148.24, 130.11, 128.60, 125.89, 20.47, 16.31; IR: ν (cm^-1^) 2926, 1761, 1476, 1370, 1215, 1166, 910, 770.

*Benzene-1,3-diyl diacetate* (**3m**) [46]: Oil. ^1^H-NMR: δ 7.35 (t, *J* = 8.0 Hz, 1H), 6.98 (d, *J* = 8.0 Hz, 2H), 6.92 (t, *J* = 2.0 Hz, 1H), 2.27 (s, 6H); ^13^C-NMR: δ 168.97, 151.13, 129.68, 118.97, 115.46, 21.06; IR: ν (cm^-1^) 3077, 2939, 1769, 1600, 1486, 1371, 1199, 1125, 1015, 915.

*2-Naphthyl acetate* (**3n**) [44]: Oil. ^1^H-NMR: δ 7.87-7.79 (m, 3H), 7.56 (s, 1H), 7.49-7.46 (m, 2H), 7.25-7.22 (m, 1H), 2.36 (s, 3H); ^13^C-NMR: δ 169.72, 148.27, 133.72, 131.44, 129.43, 127.76, 127.64, 126.56, 125.72, 121.12, 118.54, 21.23; IR: ν (cm^-1^) 3059, 2926, 1756, 1599, 1512, 1373, 1217, 1154, 761.

*Quinolin-8-yl acetate* (**3o**) [48]: Oil. ^1^H-NMR: δ 8.89 (d, *J* = 2.0 Hz, 1H), 8.13-8.10 (m, 1H), 7.69-7.65 (m, 1H), 7.52-7.35 (m, 3H), 2.50 (s, 3H); ^13^C-NMR: δ 169.86, 150.51, 147.37, 141.19, 136.09, 129.53, 126.23, 125.98, 121.77, 121.55, 21.09; IR: ν (cm^-1^) 3042, 2935, 1764, 1598, 1500, 1368, 1208, 790.

*S-Phenyl thioacetate* (**3p**) [49]: Oil. ^1^H-NMR: δ 7.41 (s, 5H), 2.41 (s, 3H); ^13^C-NMR: δ 194.19, 134.52, 129.52, 129.28, 127.91, 30.29; IR: ν (cm^-1^) 3061, 2923, 1709, 1583, 1478, 1441, 1353, 1114, 950, 747.

*S-n-Butyl thioacetate* (**3q**) [50]: Oil. ^1^H-NMR: δ 2.87 (t, *J* = 7.6 Hz, 2H), 2.32 (s, 3H), 1.59-1.52 (m, 2H), 1.43-1.34 (m, 2H), 0.92 (t, *J* = 7.2 Hz, 3H); ^13^C-NMR: δ 196.04, 31.56, 30.61, 28.82, 21.93, 13.58; IR: ν (cm^-1^) 2961, 2874, 1694, 1465, 1354, 1136, 913, 735.

*S-4-Chlorophenyl thioacetate* (**3r**) [51]: Oil. ^1^H-NMR: δ 7.40 (d, *J* = 8.4 Hz, 2H), 7.35 (d, *J* = 8.4 Hz, 2H), 2.44 (s, 3H); ^13^C-NMR: δ 193.50, 135.85, 135.72, 129.50, 126.34, 30.27; IR (film): ν (cm^-1^) 3087, 2923, 1707, 1574, 1477, 1353, 1093, 952, 821.

*S-4-Methylphenyl thioacetate* (**3s**) [51]: Oil. ^1^H-NMR: δ 7.28 (d, *J* = 8.0 Hz, 2H), 7.20 (d, *J* = 8.0 Hz, 2H), 2.37 (s, 3H), 2.35 (s, 3H); ^13^C-NMR: δ 194.53, 139.72, 134.47, 130.08, 124.54, 30.11, 21.36; IR: ν (cm^-1^) 3025, 2923, 1708, 1598, 1494, 1352, 1117, 1094, 950, 807.

## Conclusions

In summary, we have described the ruthenium-catalyzed room temperature acylation of alcohols, phenols, and thiols with acetic anhydride in an ionic liquid. Easy product isolation, facile recycling of the ionic liquid and catalyst, and avoiding use of easily volatile acetonitrile as solvent are important advantages of the developed methodology.
